# Establishment of a Novel Murine Model of Ischemic Cardiomyopathy with Multiple Diffuse Coronary Lesions

**DOI:** 10.1371/journal.pone.0070755

**Published:** 2013-08-12

**Authors:** Hajime Nakaoka, Yumiko Nakagawa-Toyama, Makoto Nishida, Takeshi Okada, Ryota Kawase, Taiji Yamashita, Miyako Yuasa-Kawase, Kazuhiro Nakatani, Daisaku Masuda, Tohru Ohama, Takashi Sonobe, Mikiyasu Shirai, Issei Komuro, Shizuya Yamashita

**Affiliations:** 1 Department of Cardiovascular Medicine, Osaka University Graduate School of Medicine, Suita, Osaka, Japan; 2 Health Care Center, Osaka University, Toyonaka, Osaka, Japan; 3 Department of Cardiac Physiology, National Cerebral and Cardiovascular Center Research Institute, Suita, Osaka, Japan; 4 Department of Community Medicine, Osaka University Graduate School of Medicine, Suita, Osaka, Japan; Albert Einstein College of Medicine, United States of America

## Abstract

**Objectives:**

Atherosclerotic lesions of the coronary arteries are the pathological basis for myocardial infarction and ischemic cardiomyopathy. Progression of heart failure after myocardial infarction is associated with cardiac remodeling, which has been studied by means of coronary ligation in mice. However, this ligation model requires excellent techniques. Recently, a new murine model, HypoE mouse was reported to exhibit atherogenic Paigen diet-induced coronary atherosclerosis and myocardial infarction; however, the HypoE mice died too early to make possible investigation of cardiac remodeling. Therefore, we aimed to modify the HypoE mouse model to establish a novel model for ischemic cardiomyopathy caused by atherosclerotic lesions, which the ligation model does not exhibit.

**Methods and Results:**

In our study, the sustained Paigen diet for the HypoE mice was shortened to 7 or 10 days, allowing the mice to survive longer. The 7-day Paigen diet intervention starting when the mice were 8 weeks old was adequate to permit the mice to survive myocardial infarction. Our murine model, called the “*modified HypoE mouse*”, was maintained until 8 weeks, with a median survival period of 36 days, after the dietary intervention (male, n = 222). Echocardiography demonstrated that the fractional shortening 2 weeks after the Paigen diet (n = 14) significantly decreased compared with that just before the Paigen diet (n = 6) (31.4±11.9% vs. 54.4±2.6%, respectively, *P*<0.01). Coronary angiography revealed multiple diffuse lesions. Cardiac remodeling and fibrosis were identified by serial analyses of cardiac morphological features and mRNA expression levels in tissue factors such as MMP-2, MMP-9, TIMP-1, collagen-1, and TGF-β.

**Conclusion:**

Modified HypoE mice are a suitable model for ischemic cardiomyopathy with multiple diffuse lesions and may be considered as a novel and convenient model for investigations of cardiac remodeling on a highly atherogenic background.

## Introduction

Heart failure is a major cause of death in developed countries. It is a common disease; more than 2% of the United States population, or almost 5 million people, are affected, and 30% to 40% of patients die within 1 year of receiving the diagnosis of heart failure. The causes of heart failure are divided into 2 main classes, ischemic and nonischemic. Coronary heart disease, the single largest cause of cardiovascular disease, is the narrowing of arteries over time caused by atherosclerotic plaques or by acute occlusion of the coronary artery by thrombosis, both of which can lead to myocardial infarction (MI) and the eventual development of heart failure [1], [2]. Today, progress in the treatment of acute MI, including reperfusion therapy by balloon catheter-facilitated vessel dilatation or pharmacological thrombolysis, coronary care units, ACE inhibitors [3], and beta blockers, enables many people to survive the acute episode. However, despite the progress in acute-phase treatment, survivors often have critical heart failure. In other words, ischemic cardiomyopathy (ICM), caused by MI and subsequent cardiac remodeling, is an unsolved problem and a significant target for medical treatment.

Murine models are very useful and important in the investigation of new treatments. Permanent or temporary occlusion of the left main descending coronary artery by coronary ligation is performed in murine models of MI or ischemic injury [4], [5]. However, the ligation model requires excellent techniques and anesthesia. Consequently, large-scale experiments are very difficult, and artificial effects from the operation and anesthesia cannot be avoided. Moreover, the most important problem is that the coronary ligation model does not have atherosclerotic lesions. On the other hand, the standard murine models for atherosclerosis, LDL receptor knockout (KO) [6] and apolipoprotein E (apoE) KO mice [7], [8], [9], which exhibit atherosclerotic lesions in the aorta, do not usually develop MI.

Braun et al. reported a murine model, HDL receptor scavenger receptor class B type I (SR-BI)-deficient and apoE-deficient double knockout mice, that exhibit coronary lesions, multiple MIs, and cardiac dysfunction. This model had very strong atherogenicity, and all of these mice died by 8 weeks of age (50% mortality: 6 weeks) [10]. Zhang et al. reported a new murine model, SRBI-deficient and hypomorphic apoE (ApoeR61^h/h^) mice, called “HypoE mice” that exhibited diet-induced hypercholesterolemia, coronary atherosclerosis, MI, and premature death (50% mortality: 33±4.9 days) [11]. In the current study, we modified the period of the atherogenic diet in HypoE mice (modified HypoE mice) so that the mice would survive MI. We now report modified HypoE mice as a new murine model of ischemic cardiomyopathy that shows multiple MIs and survives with cardiac dysfunction.

## Methods

### Animals and diets

The study was performed under the supervision of the Animal Research Committee of Osaka University and in accordance with the Japanese Act on Welfare and Management of Animals. The protocol was approved by the Animal Care and Use Committee of the Osaka University Graduate School of Medicine (Permit Number: 21-084-2). At various time points, mice were euthanized with pentobarbital (120 mg/kg intraperitoneally [i.p.]) for collection of tissue and blood samples. For pain management during coronary angiography (CAG), mice were anesthetized with inhaled isoflurane (4–5% for induction and 2–3% for maintenance).

SR-BI KO/ApoeR61h/h mice (mixed C57BL/6×129 background) were obtained as a gift from Monty Krieger, Biology Department, Massachusetts Institute of Technology, USA. These mice were housed and fed a normal chow diet from weaning to 8 weeks of age in our breeding laboratory. Male mice were weaned at 21 to 27 days of age and 2 or 3 mice per cage were housed in a barrier facility under specific pathogen-free condition with a 12-hour light/12-hour dark cycle. Beginning at the age of 8 weeks, the mice were fed the Paigen diet for 7 or 10 days (dietary intervention); the Paigen diet was then replaced with normal chow.

The Paigen diet, which contained 7.5% cocoa butter, 1.25% cholesterol, and 0.5% sodium cholate, was prepared at Oriental Yeast Co, Ltd, Tokyo. The caloric composition of this diet was 21.4% protein, 27.4% fat, and 51.2% carbohydrate. The original Paigen diet was described previously [12], [13], [14]. In contrast, the caloric composition of the normal chow diet (MF diet, Oriental Yeast Co, Ltd.) was 25.6% protein, 12.8% fat, and 61.6% carbohydrate.

The findings of previous studies on SR-BI KO mice showed that, female, but not male, SR-BI KO/ApoeR61^h/h^ mice are infertile. Thus, female ApoeR61^h/h^ mice with heterozygous null mutations in SR-BI were mated to male SR-BI KO/ApoeR61^h/h^ mice [11]. The genotypes were determined by polymerase chain reaction as previously described [15], [16].

### Cardiac functional analysis

Noninvasive measurements of blood pressure were performed on in the mice, using a blood pressure monitor for rats and mice (Model BP98-A, Softron Co., Ltd., Tokyo, Japan) according to the manufacturer’s instructions. To perform echocardiography on conscious mice, ultrasonography (Vevo770, VisualSonics, Inc., Toronto, Canada) was performed using a 25 MHz linear transducer (VisualSonics). The heart was imaged in the 2-dimensional parasternal long-axis view, and an M-mode echocardiogram of the midventricle was recorded at the level of the papillary muscles.

### Morphological and biochemical analysis

The heart and ascending aorta of the mouse were perfused with phosphate-buffered saline (PBS) containing 1% heparin, via the left ventricular apex. Samples were isolated and fixed with formalin. Paraffin sections (10 μm) of hearts were stained with Masson’s trichrome (Sigma -Aldrich, St. Louis, USA) to evaluate fibrotic areas. The distributions of fibrosis in the middle ventricle and apex were examined with respect to each compartment as described in the previous report [17]. The percent fibrosis was evaluated in 7 sections in different locations as follows: section 1, upper septum; 2, anterior left ventricular wall; 3, upper lateral left ventricular wall; 4, posterior left ventricular wall; 5, right ventricular wall; 6, lower septum; and 7, lower lateral left ventricular wall. Sections 1–5 were located in the middle ventricle and sections 6–7 in the apex.

Frozen sections of ascending aortas at the level of the aortic valve were stained with oil red O and hematoxylin. The sizes of the atherosclerotic lesions were calculated as the sum of the cross-sectional areas of oil red O positive-stained plaques, using Image J software.

Blood was collected from the *ad libitum* fed mice at the time of sacrifice with an overdose of pentobarbital. Serum was separated by centrifugation and stored at −80°C. Serum concentrations of triglyceride, insulin, creatinine, and glucose were determined using commercially available enzymatic assay kits according to the manufacturers’ instructions. At each evaluation point, the lipid profile was examined using an HPLC (high performance liquid chromatography) method as previously described [18], [19].

### Real-time reverse transcription polymerase chain reaction

Real-time reverse transcription polymerase chain reaction (RT-PCR) was performed according to the manufacturer’s protocol (SuperScript VILO cDNA Synthesis Kit, Life Technologies Co., Carlsbad, USA). The total RNA was prepared from hearts at various time points after surgery. Total RNA was extracted from snap-frozen, homogenized tissue from the left and right ventricles. RNA was DNase-treated using SuperScript VILO and reverse-transcribed using the QuantiTect Reverse Transcription Kit (QIAGEN, Hilden, Germany). RT-PCR was performed using the Universal Probe Library (UPL) (Roche, Basel, Switzerland) and Light Cycler TaqMan Master kit (Roche). Relative levels of gene expression were normalized to the level of mouse GAPDH expression using the comparative Ct (Threshold Cycle) method according to the manufacturer’s instructions [20].

### Coronary angiography

Mice were anesthetized, intubationed, and heparinized. A catheter was inserted from the right carotid artery into the ascending aorta, and a solution consisting of 50% weight/volume barium sulfate suspended in 7% gelatin (weight/volume solution in water warmed in a water bath to 60°C) was injected into the ascending aorta. The heart of each mouse was then harvested and immersed in ice to solidify the contrast agent. Coronary angiography was performed using an angiographic system (MFX-80HK, Hitex Co, Ltd., Osaka, Japan) consisting of an open-type 1 μm microfocus X-ray source (L9191, Hamamatsu Photonics Co, Ltd., Hamamatsu, Japan) and a 50/100 mm (2 inch/4 inch) dual mode X-ray image intensifier (E5877JCD1-2N, Toshiba Co, Ltd., Tokyo, Japan) at 60 kV and 60 μA.

### Statistical analysis

Results are shown as mean ± S.E. Paired data were evaluated using Student’s *t*-test. A 1-way analysis of variance with Tukey's multiple comparison test was used for multiple comparisons. The Kaplan-Meier method with a log-rank test was used for survival analysis.

A *p* value <0.05 for differences was considered statistically significant.

## Results

### Hypo E mice survived 7 but not 10 days on the Paigen diet

**Figure 1:**
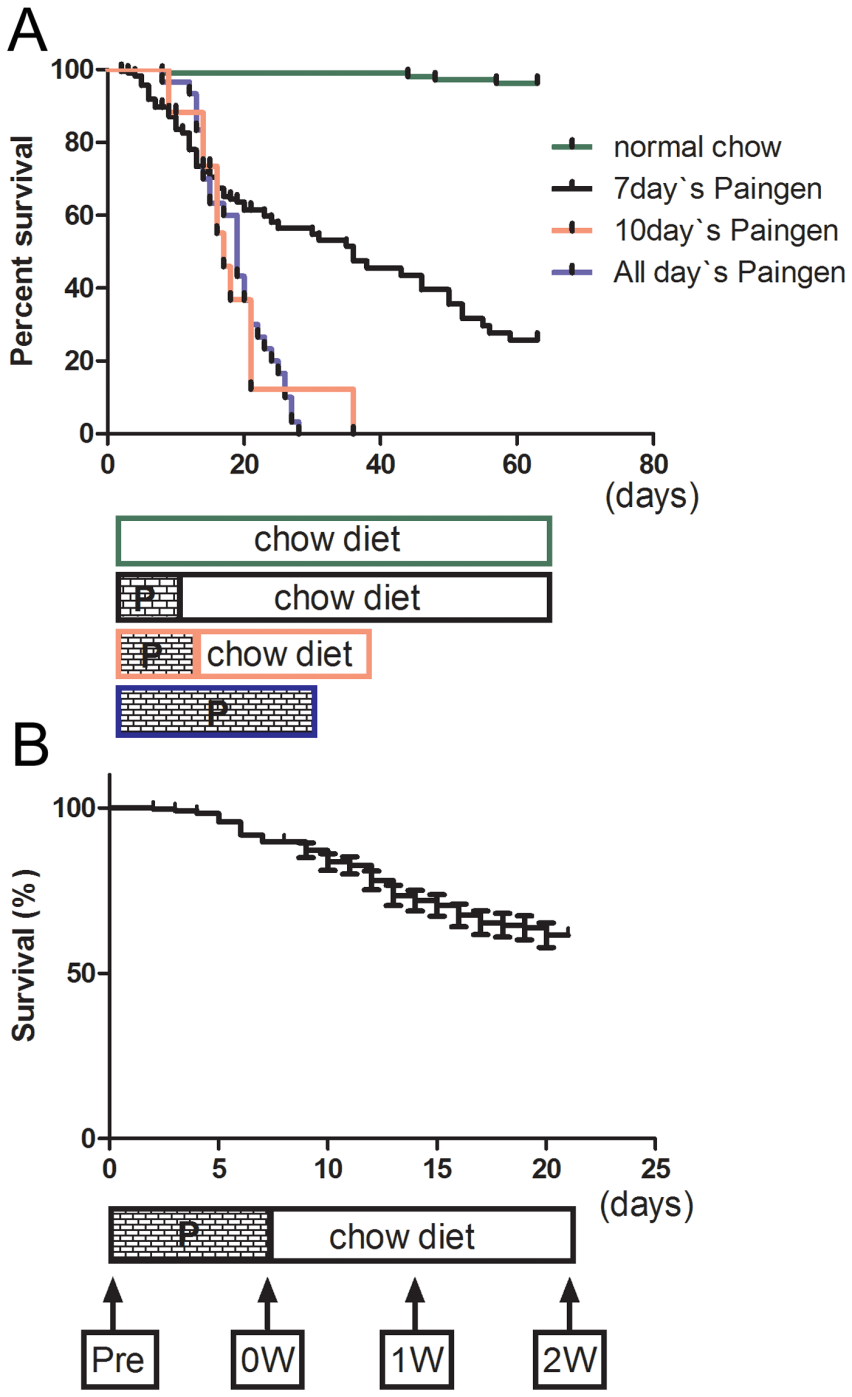
Effects of the Paigen diet on the survival rate of HypoE mice. Survival curves of HypoE mice observed for various periods of the Paigen diet intervention (A). Survival curve of HypoE mice after 7 days on the Paigen diet and timing of observations are shown (B). P shows the Paigen diet. Black line, 7-day Paigen diet intervention; red line, 10-day intervention; blue line, continuous feeding of Paigen diet. Pre, just before Paigen diet; 0W, at the end of the 7-day Paigen diet intervention; 1W, 1 week after the end of the 7-day Paigen diet intervention; 2W, 2 weeks after the end of the 7-day Paigen diet intervention.

Very few HypoE mice consuming the normal chow diet died during the experimental period (**Fig. 1**). Long-term observation of HypoE mice consuming a normal chow diet showed a median survival time of 192 days. We found that these HypoE mice died from MI or heart failure rather than from cerebral infarction. The atherogenic Paigen diet caused early death in HypoE mice, and most mice died within 1 month (**Fig. 1A**, blue line). It was expected that HypoE mice could survive on the Paigen diet for a short time. However, 8-week-old HypoE mice fed the Paigen diet for 10 days demonstrated a similar survival curve (**Fig. 1A**, red line) to that for those fed the Paigen diet continuously. In our breeding laboratory, HypoE mice survived after 7 days on the Paigen diet. We called these mice “modified HypoE mice.” Their median survival period was 36 days after the Paigen diet intervention. The survival rate of the modified HypoE mice fell rapidly during the first 20 days and slowly thereafter (**Fig. 1A**, black line). Therefore, the observation period of the current study was 2 weeks after the end of the 7-day Paigen diet intervention. At the end of this period, 67% of the modified HypoE mice remained alive. All of the dead mice were dissected, and MI scars and enlarged hearts were found in all cases. Modified HypoE mice were examined just before beginning the Paigen diet (Pre), after 7 days on the Paigen diet (0W), 1 week after the end of the 7-day Paigen diet intervention (1W), and 2 weeks after the end of the 7-day Paigen diet intervention (2W) (**Fig. 1B**).

### The atherogenic Paigen diet temporally increased cholesterol levels

The Paigen diet has very strong atherogenicity. The effects of the Paigen diet on the cardiovascular risk profiles of modified HypoE mice were confirmed (**Table 1**). The body weights of the mice did not change during the Paigen diet intervention but gradually increased afterward. The blood pressure and blood glucose level increased slightly but not significantly after the Paigen diet. The creatinine level and heart rate did not show clear trends. The insulin level temporally increased during the atherogenic diet. Notably, the serum levels of total cholesterol, LDL-C, Chylomicron-C and VLDL-C increased markedly after the Paigen diet intervention and rapidly returned to the pretreatment levels 1 week after the end of Paigen diet intervention. The HDL-C levels, in contrast, decreased permanently after the Paigen diet intervention.

**Table 1:** Paigen diet-induced changes in modified HypoE mice.

	Pre	0 week	1 week	2 weeks
Body weight (g)	26.6±2.7	26.2±2.3	27.1±2.6	28.1±2.6*
SBP (mmHg)	91±6	91±6	96±4	108±24
DBP (mmHg)	52±6	59±4	59±11	58±9
Heart rate (beats/min)	637±26	620±31	645±32	595±81
Blood glucose (mg/dL)	131±27	164±8	166±34	143±44
Insulin	53.9±31.1	82.9±89.6	42.3±26.6	45.8±22.1
Creatinine (mg/dL)	0.44±0.13	0.42±0.09	0.45±0.08	0.50±0.13
Total cholesterol (mg/dL)	318±89	873±90^***^	347±13	361±50
Chylomicron-C(mg/dL)	56±15	293±34^***^	68±3	68±15
VLDL-C(mg/dL)	159±57	415±47^***^	191±8	185±22
LDL-C (mg/dL)	74±22	134±10^***^	75±8	89±15
HDL-C (mg/dL)	28±6	30±4	13±1^***^	20±4**

SBP, systolic blood pressure; DBP, diastolic blood pressure.

Data are mean ± SD.

* *P*<0.05, **P<0.005, ***P<0.0001. Compared with Pre-diet intervention period.

### An atherogenic diet rapidly induced atherosclerotic changes in modified HypoE mice

**Figure 2:**
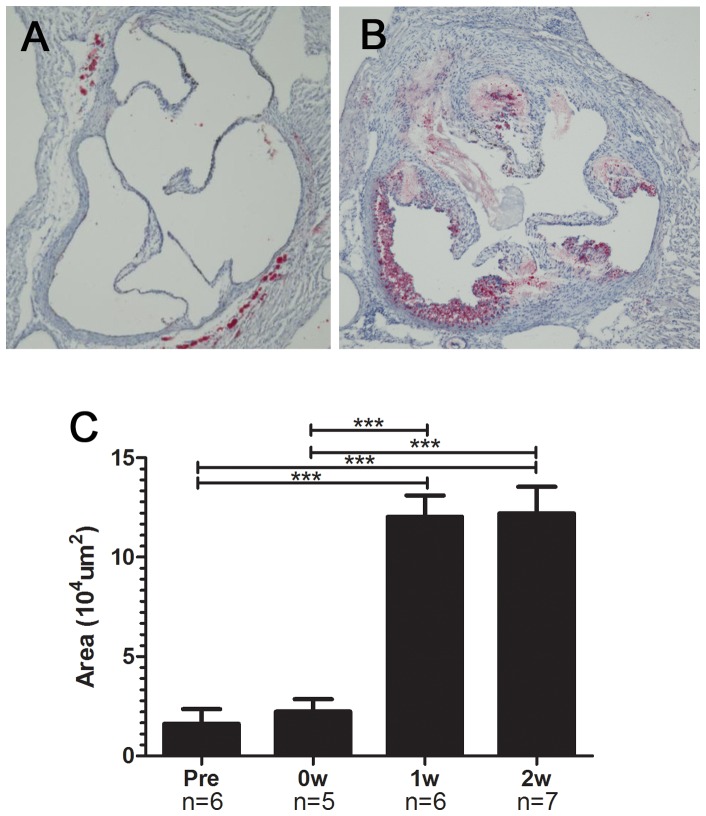
Atherosclerotic lesions in the aorta of modified HypoE mice. Atherosclerotic lesions at the level of the aortic valve were evaluated by oil red O staining. Representative photographs of specimens taken just before the Paigen diet (A) and 2 weeks after the end of the 7-day Paigen diet intervention (B) are shown. Area of atherosclerotic lesions markedly increased 1 week after the end of the 7-day Paigen diet intervention (C). Pre, 0W, 1W, and 2W represent the same time points as in Figure****1. ***P<0.001.

**Figure 3:**
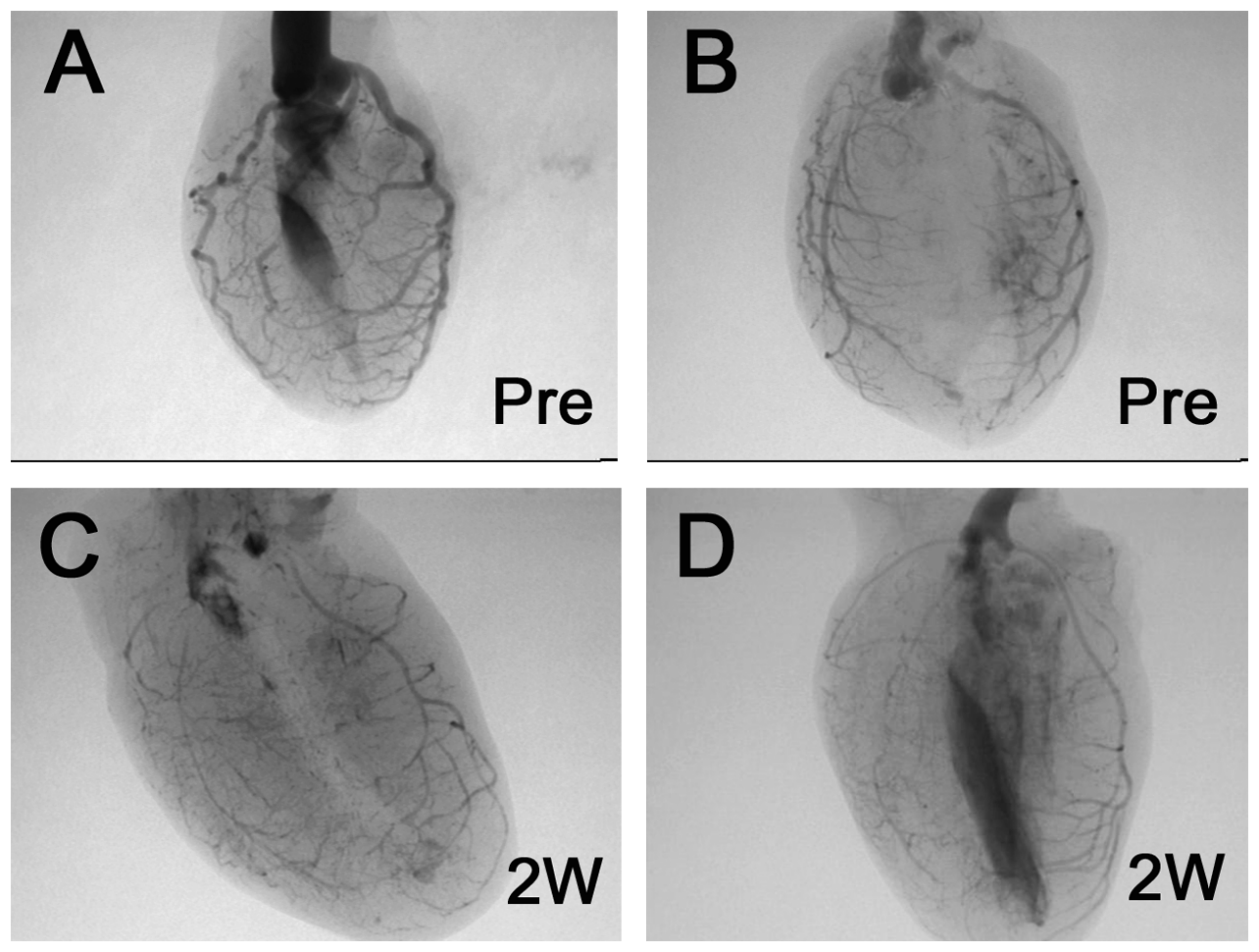
Coronary angiography of modified HypoE mice. The coronary arteries of HypoE mice just before the Paigen diet (A) and 2 weeks after the end of the 7-day Paigen diet intervention (B).

The total area of the atherosclerotic lesions in the aortic roots was markedly increased after the Paigen diet (**Fig. 2** **, A and B**). A quantitative analysis of the atherosclerotic lesions is shown in **Figure 2C**. The plaque area in modified HypoE mice 1 week after the Paigen diet was 5.4 times larger than that just before the Paigen diet. There was no significant increase in lesion area between 1 week and 2 weeks after the dietary intervention. Coronary angiography (CAG) showed multiple diffuse lesions (**Fig. 3**). Branches from the main coronary artery were rarely evaluated because of the low resolution of the images. Therefore, even successful CAG was able to detect occlusion only of the main coronary arteries. Five CAG procedures in pre-treatment mice revealed no main coronary occlusion, whereas CAG of 6 modified HypoE mice detected 2 occlusions of the RCA, 2 of the septal artery, and 1 of the LCA.

### Cardiac fibrosis and cardiac function after the atherogenic diet

**Figure 4:**
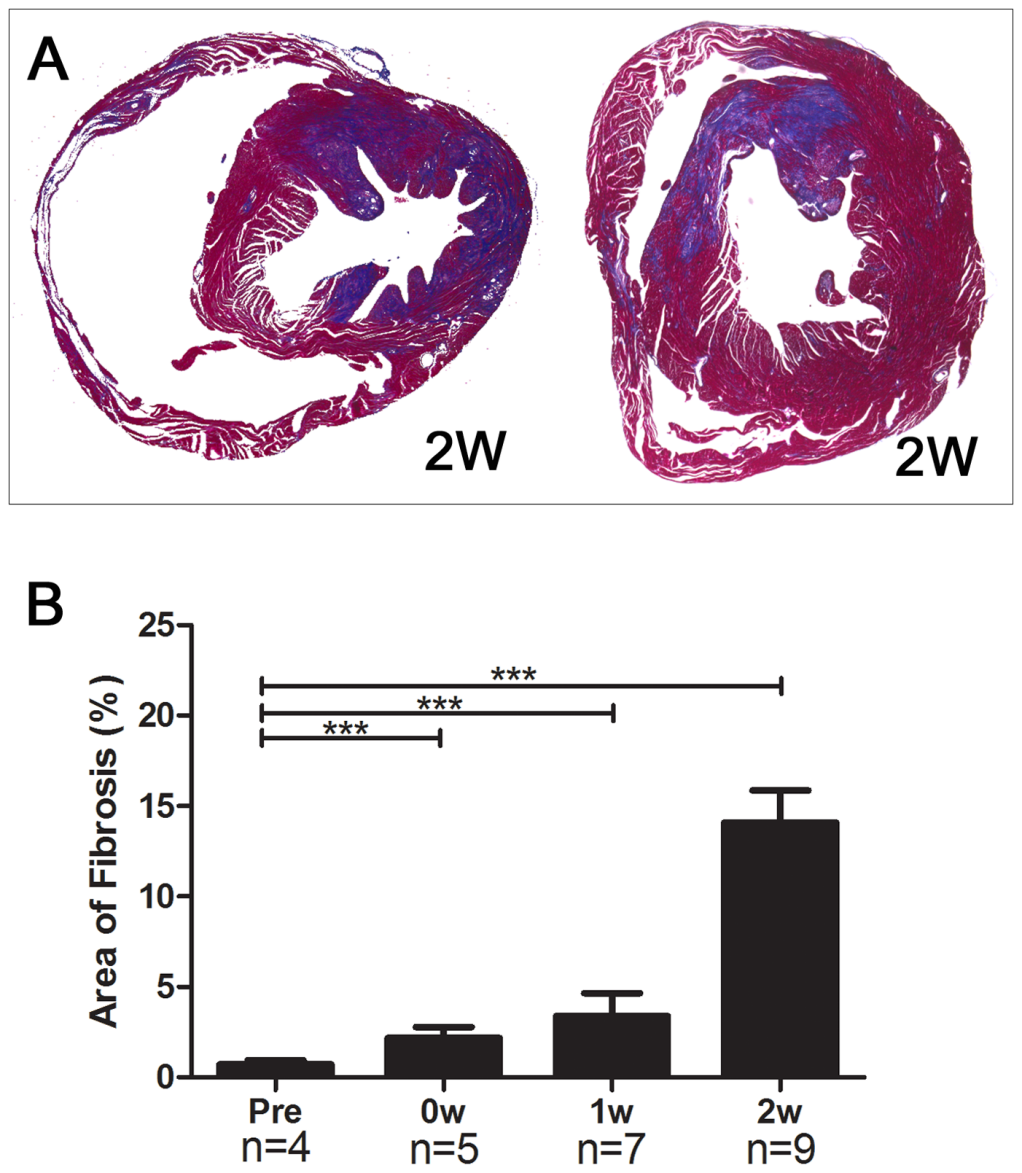
Cardiac fibrosis in modified HypoE mice. Representative images of cardiac fibrosis stained with Masson’s trichrome 2 weeks after the end of the 7-day Paigen diet intervention (A) and the percent cardiac fibrosis as a function of time. (B). Pre, 0W, 1W, and 2W represent the same time points as in Figure****1. ***P<0.001.

Cardiac fibrosis was observed in all of the HypoE mice after the atherogenic Paigen diet intervention. Fibrotic lesions were patchy and were located predominantly near the endocardium (**Fig. 4A**).

Quantitative analysis of cardiac fibrosis was performed using Masson’s trichrome staining (**Fig. 4B**). Cardiac fibrosis was particularly increased 2 weeks after the Paigen diet intervention. The extent of cardiac fibrosis in modified HypoE mice 2 weeks after the Paigen diet intervention ranged from 5.3% to 22.3%. At this time, the echocardiographic parameters LVDd and Fractional shortening (FS) ranged from 3.6 mm to 5.6 mm and from 11.7% to 45.5%, respectively. The ventricular fibrosis was not predominantly in any one location, and there was no significant difference in fibrosis between the middle ventricle and apex (**Table 2**). Areas 3 and 7 are perfused by the same main coronary artery, as are areas 1 and 6. However, we observed no significant relationship either between the percent fibrosis levels of areas 3 and 7 or between those of areas 1 and 6.

**Table 2:** Distribution of percent fibrosis in the various parts of middle ventricle and apex.

								Middle	Apex
	Middle ventricle				Apex			Area 1–4	Area 5–7
Mouse	area1	area2	area3	area4	area5	area6	area7	Total	Total
1	4.1	13.2	2.6	2.7	4.9	22.9	2.3	5.3	10.3
2	17.3	14.1	9.7	11.3	12.0	15.9	43.7	13.5	22.1
3	8.0	53.0	23.8	27.3	19.0	37.2	2.9	22.3	10.8
4	18.9	8.7	6.2	6.0	7.8	4.2	5.8	10.5	6.3
8	29.2	18.0	10.6	11.8	9.7	81.0	7.3	17.3	29.0
6	25.7	12.3	5.5	4.4	5.5	24.6	4.0	11.7	9.4
7	53.7	5.1	2.4	2.7	13.2	13.4	2.2	17.9	11.5
8	3.7	1.9	10.8	33.2	2.0	7.1	2.5	9.8	4.6
9	10.5	50.7	9.0	10.8	1.7	17.5	21.3	18.6	18.2
**Mean**	19.0	19.7	9.0	12.2	8.4	24.9	10.2	14.1	13.6
**±SD**	±15.8	±18.9	±6.4	±10.9	±5.6	±23.2	±13.9	±5.3	±8.0

Data are expressed as percentage of fibrosis area.

Area 1, upper septum; area2, anterior left ventricular wall; area3, upper lateral left ventricular wall; area4, posterior left ventricular wall; area5, right ventricular wall; area6, lower septum; and area7, lower lateral left ventricular wall. Areas 1–5 were located in the middle ventricle and areas 6–7 in the apex.

**Figure 5:**
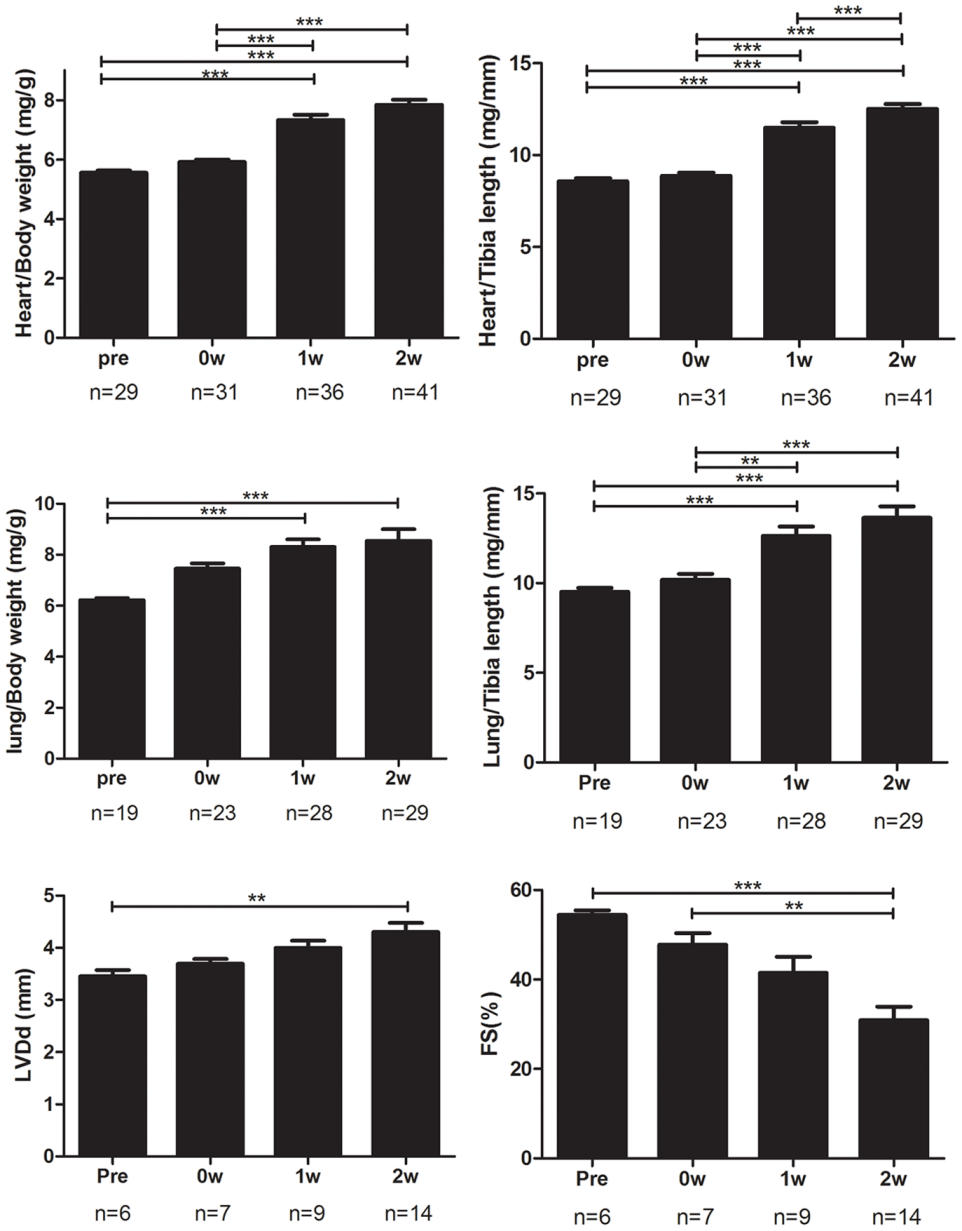
Signs of heart failure and cardiac function. Signs of heart failure, which manifested as increases in the heart and lung weights adjusted by body weight or tibia length, were observed. Echocardiography demonstrated an increase in the left ventricular end-diastolic dimension (LVDd) and a decrease in fractional shortening (FS) after the Paigen diet intervention. Pre, 0W, 1W, and 2W represent the same time points as in Figure****1. *P<0.05, **P<0.01, ***P<0.001.

**Figure 6:**
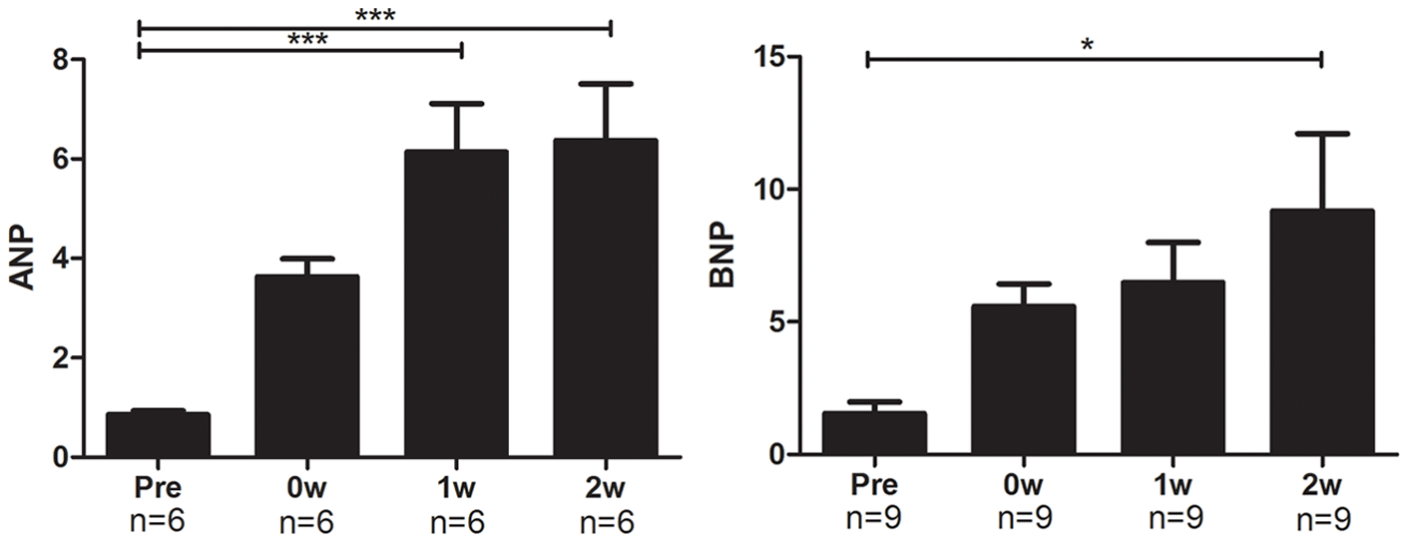
Expressions of ANP and BNP in the hearts of modified HypoE mice. The expression levels of the markers of heart failure, ANP and BNP, gradually increased from the end of the 7-day Paigen diet intervention. ANP, atrial natriuretic peptide; BNP, brain natriuretic peptide. Pre, 0W, 1W, and 2W represent the same time points as in Figure****1. *P<0.05, ***P <0.001.

Signs of heart failure which manifested as increases in the heart weight and lung weight were gradually evident from 1 week after the dietary intervention. Echocardiography revealed a significant decrease in cardiac function (**Fig. 5**). FS 2 weeks after the Paigen diet (n = 14) was significantly decreased as compared with that just before the Paigen diet (n = 6) (31.4±11.9% vs. 54.4±2.6%, respectively, *P*<0.01). The expression levels of atrial natriuretic peptide (ANP) and brain natriuretic peptide (BNP) in the heart also showed the progression of heart failure (**Fig. 6**).

### Changes in gene expression related to cardiac remodeling were evident after the atherogenic diet intervention

**Figure 7:**
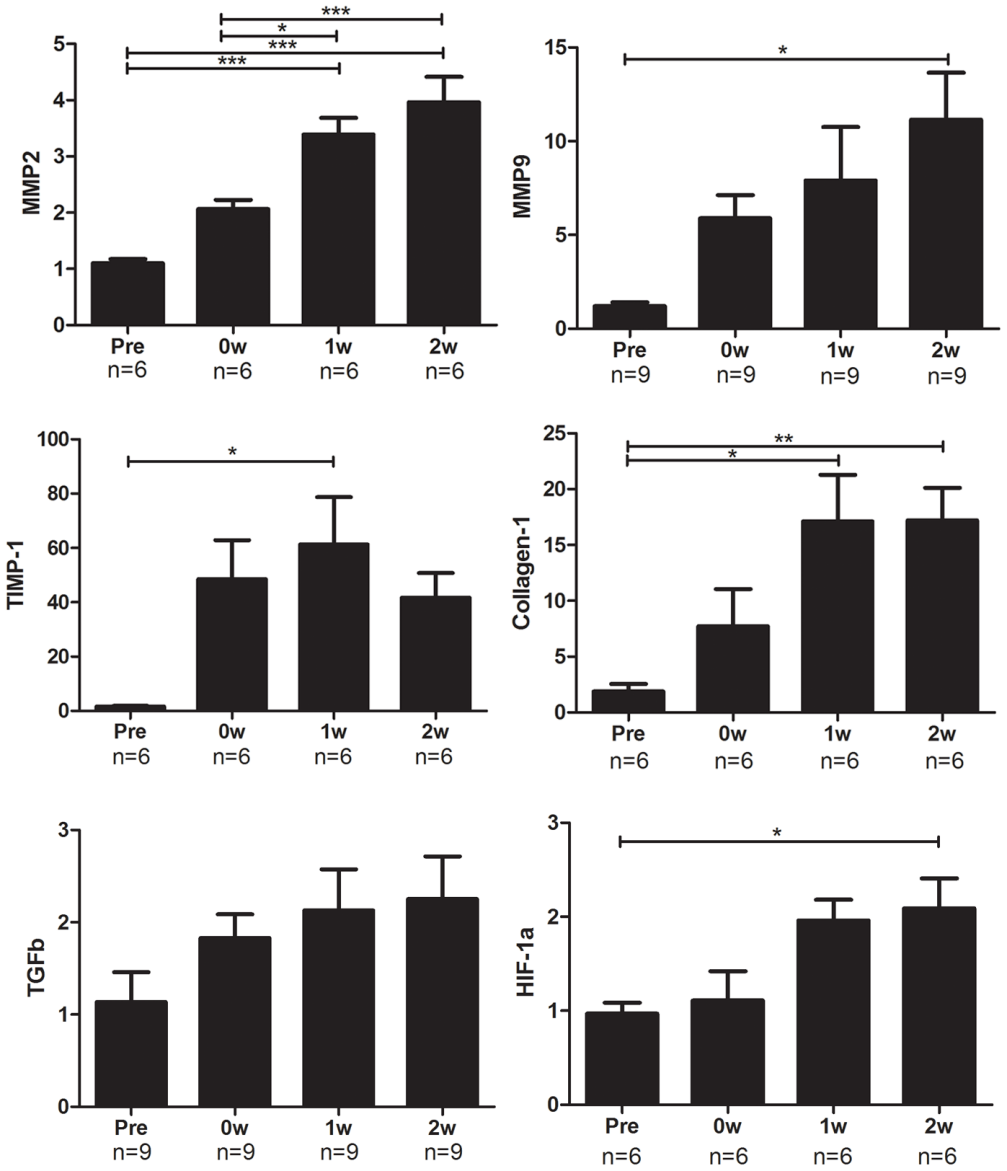
mRNA expression levels of remodeling-related genes and HIF-1αin the hearts of modified HypoE mice. The expression levels of MMP-2, MMP-9, TIMP-1, Collagen-1, and TGF-β show that cardiac remodeling was induced. HIF-1α was also induced along with these mRNA expressions. MMP, matrix metalloproteinase; TIMP, tissue inhibitor of metalloproteinase; TGF, transforming growth factor; HIF, hypoxia-inducible factor. Pre, 0W, 1W, and 2W represent the same time points as in Figure****1. *P<0.05, **P<0.01, ***P<0.001.

The changes in mRNA expression levels of genes related to cardiac remodeling are shown in **Figure 7**. The expression levels of matrix metalloproteinase (MMP)-2 and tissue inhibitor of metalloproteinase (TIMP)-1 in the heart gradually increased from pretreatment to 2 weeks after the dietary intervention. The expression levels of MMP-9 and collagen-1 also increased from their pretreatment levels, although there was no increase between 1 week and 2 weeks after the Paigen diet intervention. Transforming growth factor (TGF)-β and hypoxia-inducible factor (HIF)-1α are important molecules that regulate cardiac remodeling. The expression levels of these genes showed a tendency to increase after the dietary intervention.

## Discussion

### Advantages of HypoE mice as an MI model

To establish the murine model of ICM, we employed HypoE mice as an MI model, modified the period of atherogenic diet feeding, and successfully identified the characteristics of ICM. The MI model most often used is the coronary ligation model, in which MI is induced by the ligation of the left anterior descending coronary artery (LAD) [4], [5], [21]. Ligation-model mice are also utilized to investigate ICM after acute MI [22]. The use of ligation-model mice has revealed many new pathological and physiological pathways involved in MI and identified proteins associated with MI [23]. The ligation model usually require anesthesia, intubation, and thoracotomy, which induce artificial injuries and inflammation at sites other than the area of MI [24]. To avoid these artificial effects, the ligation model has been improved. In the “closed chest’’ method, a suture or occlusion device is implanted for ligation of the LAD via thoracotomy. Using the device, the LAD is ligated from outside the body several days after the operation to prevent acute artificial effects of the surgery [25], [26]. Gao et al. reported another improved ligation method in which the heart was manually exposed without intubation through a small incision [27]. This method shortens the operation time and minimizes the operative stress, but it requires excellent techniques. Despite these improvements to the ligation method, the model continues to pose substantial problems.

Human patients with MI often have risk factors such as hypertension, dyslipidemia, and glucose intolerance, which impair endothelial function and induce coronary atherosclerosis at multiple sites. These patients sometimes have multiple coronary lesions and multiple ischemic lesions. In contrast, the ligation-model mice do not have atherosclerotic lesions, and their endothelial function is intact. When MI is induced in the LAD area by coronary ligation, other intact areas fully induce the compensatory reactions and pathways that protect cardiac function. In humans, multiple ischemic lesions other than the MI lesion may influence the induction of these compensatory reactions and pathways. Therefore, it is desirable to establish other murine MI models that more closely resemble the nature of MI encountered in the clinical setting.

On the other hand, murine models for atherosclerosis have been established. ApoE-deficient mice are the mouse model most often used to investigate aortic atherosclerosis, but they do not show coronary occlusion [7], [8], [9]. Feeding these mice a high-fat diet, particularly the Paigen diet, induces atherosclerotic lesions in the aorta, although not in the coronary arteries [28]. MI cannot be induced in apoE-deficient mice even by feeding the Paigen diet [29]. However, some murine MI models, most of which are genetically modified apoE-deficient mice [30], [31], have been reported. Gene modification in mice can induce MI. Akt1 and apoE deficient mice exhibit reduced migration of vascular smooth muscle cells [30]. Mice with these deficiencies are useful for investigate specific mechanisms of MI, but these generally differ from MI as encountered in the clinical settings.

HypoE mice showed high-fat diet-induced MI lesions. High-fat-fed HypoE mice demonstrate marked hypercholesterolemia and increased levels of remnant lipoproteins [11], that resemble familial hypercholesterolemia and the dyslipidemia involved with diabetes or metabolic syndrome, respectively. Further investigations using synchrotron radiation [32] may reveal impairment of coronary endothelial function in HypoE mice. We believe that the high-fat-fed HypoE mice can be a useful model to investigate MI and cardiac remodeling.

### Modification of the atherogenic Paigen diet

Recently, Toyama and Zhang reported the effects of dietary manipulation and social isolation on HypoE mice [33]. They also investigated the survival rates of HypoE mice that were fed the Paigen diet for 10, 12, or 14 days. HypoE mice survived 10 days of the Paigen diet. The authors proposed that HypoE mice might be a promising novel model for the study of heart remodeling, but they did not demonstrate other evidence of cardiac remodeling. We revealed histological changes, cardiac function, and the expression levels of cardiac remodeling-related genes for the first time in this mouse model.

In our laboratory, HypoE mice did not survive 10 days of the Paigen diet. Total cholesterol levels did not contribute to the observed difference in survival, because total cholesterol levels were higher in the previously described Paigen diet-fed HypoE mice (1600 mg/dL) than in our modified HypoE mice [13]. One possible explanation for the difference in survival is that the mice do not tolerant environmental changes. For example, social isolation reduced the survival rate of Paigen diet-fed HypoE mice [33]. These findings indicate that the appropriate duration of the Paigen diet should be determined each time HypoE mice are imported into a new breeding laboratory.

### Multiple diffuse coronary lesions in the modified HypoE mice

Histological examination of modified HypoE mice showed that the lesions of cardiac fibrosis were patchy and were predominantly located near the endocardium. This finding implies the presence of multiple distal occlusions of the coronary arteries. Moreover, the cardiac fibrosis was not distributed predominantly in any one region of the heart. The data in Table 2 suggest that the fibrosis in the ventricle was attributable not only to occlusions of the main coronary arteries but also to distal microvascular lesions. Another possible explanation is that diffuse fibrosis developed in response to repeated episodes of occlusion and reperfusion at many different sites in the coronary arteries.

CAG clearly demonstrated occlusions of the main coronary arteries 2 weeks after the Paigen diet treatment. However, these occlusions alone cannot explain the diffuse distribution of cardiac fibrosis. Although we cannot provide quantitative data, CAG seemed to show multiple and diffuse stenosis as well as a decreased number of branched small coronary arteries in modified HypoE mice. These observations suggest that distal microvascular lesions can also contribute to diffuse cardiac fibrosis.

ICM was originally defined as chronic heart failure with severe multiple coronary lesions, but it is now often defined as chronic heart failure with significant coronary lesions [34], [35]. CAD patients with diabetes or those who are elderly often demonstrate multiple diffuse lesions of the coronary arteries [36], [37]. Familial hypercholesterolemia patients with visceral fat accumulation sometimes exhibit multiple diffuse lesions [38]. Modified HypoE mice can be a useful model of ICM for investigating MI and cardiac remodeling in these clinical syndromes.

Histological examination revealed no obvious plaque rupture. Massive accumulation of foam cells in the coronary lumen was often present, and coronary occlusion by foam cells and thrombus was sometimes observed in this model (**Fig. S1**). These findings are compatible with those from previous reports on HypoE mice [11]. The thrombus formation observed was likely due to the extreme hyperlipidemia and accumulation of foam cells causing injury to the endothelial cells of the coronary arteries and thus attenuating their anti-thrombotic effects.

However, SR-BI/apoE dKO mice developed coronary artery lesions that more closely resembled human coronary lesions, with more severe and complex pathological features such as fibrin deposition [10]. Our modified HypoE mice did not develop complex lesions. However, long-term observation of modified HypoE mice might also reveal the development of complex lesions with fibrin deposition as seen in SR-BI/apoE dKO mice.

### Cardiac remodeling in the modified HypoE mice

We examined the time course of cardiac remodeling in the modified HypoE mice and successfully identified changes indicative of cardiac remodeling. Serum levels of troponin I markedly increased just after the Paigen diet intervention and then quickly decreased but remained higher than the pre-intervention levels (data not shown). This result showed that MI was mainly induced during the 7 days of the Paigen diet intervention and that myocardial injury, probably due to mild ischemia, continued from 1 week to 2 weeks after the Paigen diet intervention ended. Serum levels of IL-6 demonstrated changes similar to those of troponin I (data not shown). The expression of MMP-9, which is mainly derived from macrophages [39], remained elevated until 1 week after the Paigen diet intervention. Therefore, macrophages may be recruited to repair the injured MI tissue in the acute phase of cardiac remodeling.

Heart failure, represented by ANP and BNP expression, was induced just after MI, but these changes were not significant. During 1 week and 2 weeks after MI, Masson’s trichrome staining, ANP and BNP expression, and echocardiography revealed significant progression of cardiac fibrosis, heart failure, and cardiac dysfunction. The expression of collagen-1 peaked 1 week after MI. Therefore, cardiac fibrosis accelerated during 1 week and 2 weeks after MI and might show a constant increase thereafter. This appears to be the cause of the continuing steady decrease in survival 2 weeks after the Paigen diet. MMP-2 is involved in neovascularization and enlargement of the left ventricle (LV) [40], [41]. The expression levels of MMP-2 and TIMP-1 seem to increase in parallel with LV enlargement. The changes in the expression levels of these genes may represent cardiac remodeling in the modified HypoE mice. To investigate the cause of fibrosis, we examined TGF-β, which is an important factor in the induction of fibrosis [42], [43], and HIF-1α, which controls gene expression under ischemic conditions [44]. The expression levels of TGF-β and HIF-1α increased gradually, but did not show clear changes when compared with the expression levels of other genes. Therefore, TGF-β and HIF-1α do not appear to be the main controllers of the expression of genes related to cardiac remodeling in the modified HypoE mice. Our modified HypoE mice may be a useful model for evaluating pharmacological effects and investigating pharmacological targets in cardiac remodeling.

### Reproducibility and limitations of modified HypoE mice

The reproducibility of experimental data is very important for animal models. However, the standard deviations of FS (6%) and cardiac fibrosis (11%) measured 2 weeks after the Paigen diet intervention seem large compared with those from reports using the ligation model. Previous reports found SDs of FS of 2.4% [45] and 5.5% [46], and SD of cardiac fibrosis of approximately 5–10% [47]. The relatively low reproducibility and the wide variations of measures of cardiac function and fibrosis are probably caused by the presence of multiple coronary lesions that occlude coronary arteries at various positions determined by chance.

There are major limitations to the use of this model. First, mechanical occlusion models of MI induce a highly reproducible infarct at a specific time point; this is not the case in modified HypoE mice, in which the pathology is likely to differ with the specific pattern of coronary disease produced. Second, if clinical translation is the goal, the model does not recapitulate the pathophysiologic basis of CAD in humans (which likely involves multiple factors), as it appears to induce extremely severe hypercholesterolemia in the absence of other major coronary risks.

In conclusion, we demonstrated that 7 days on the atherogenic Paigen diet induced MI and subsequent cardiac remodeling with multiple diffuse coronary lesions in HypoE mice.

This model mouse, called the modified HypoE mouse, is well suited as a model of ischemic cardiomyopathy and may be considered as a novel and convenient model for the investigation of cardiac remodeling on a highly atherogenic background. This may be a useful murine model to evaluate pharmacological effects and to investigate pharmacological targets in MI and cardiac remodeling.

## Supporting Information

Figure S1 (TIF)
