# Correction: Establishment of a Novel Murine Model of Ischemic Cardiomyopathy with Multiple Diffuse Coronary Lesions

**DOI:** 10.1371/annotation/8d40f661-efc4-4590-8922-b3a6f5374e12

**Published:** 2013-08-27

**Authors:** Hajime Nakaoka, Yumiko Nakagawa-Toyama, Makoto Nishida, Takeshi Okada, Ryota Kawase, Taiji Yamashita, Miyako Yuasa-Kawase, Kazuhiro Nakatani, Daisaku Masuda, Tohru Ohama, Takashi Sonobe, Mikiyasu Shirai, Issei Komuro, Shizuya Yamashita

The correct version of Figure 1 is available here: 

**Figure:**
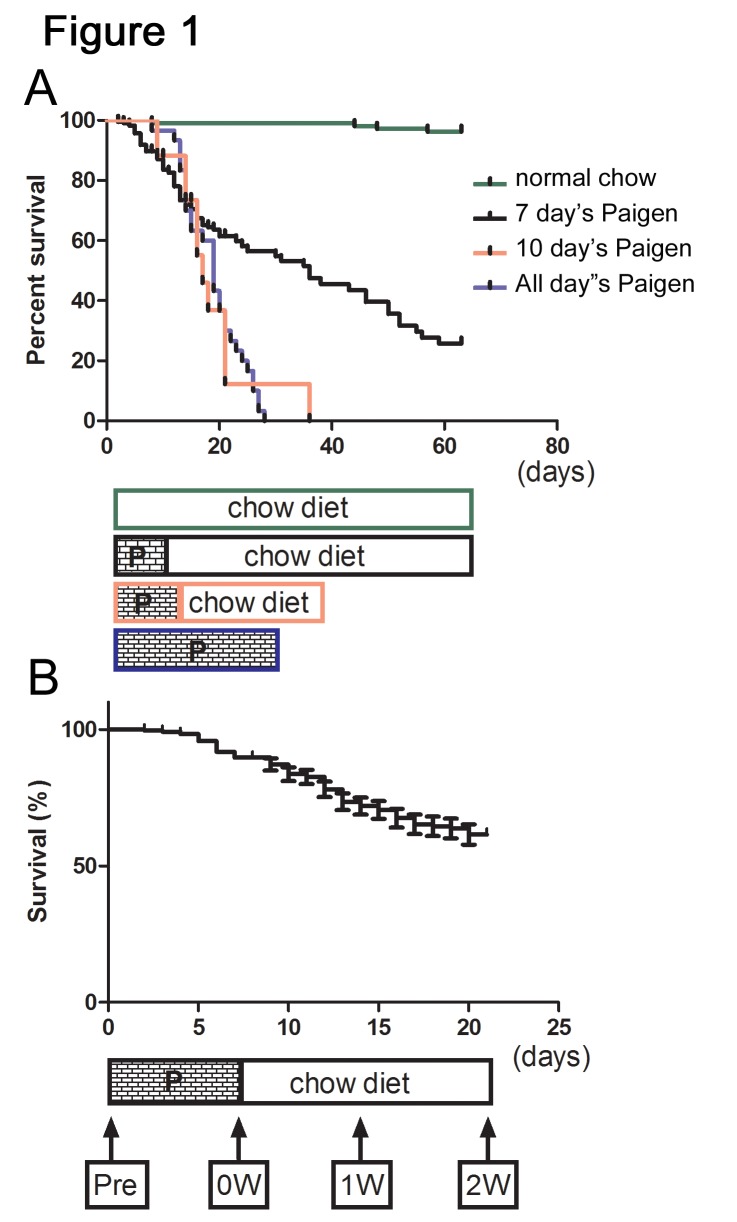


The correct version of the Figure 3 legend is available below:

**Figure 3. Coronary angiography of modified HypoE mice.**

The coronary arteries of HypoE mice just before the Paigen diet (A, B) and 2 weeks after the end of the 7-day Paigen diet intervention (C, D). 

