# Supplementary figures and images for: Establishment of a Novel Murine Model of Ischemic Cardiomyopathy with Multiple Diffuse Coronary Lesions
Source: PLoS One. 2013 Aug 12;8(8):e70755. doi: 10.1371/journal.pone.0070755 (PMC3741297; doi:10.1371/journal.pone.0070755)

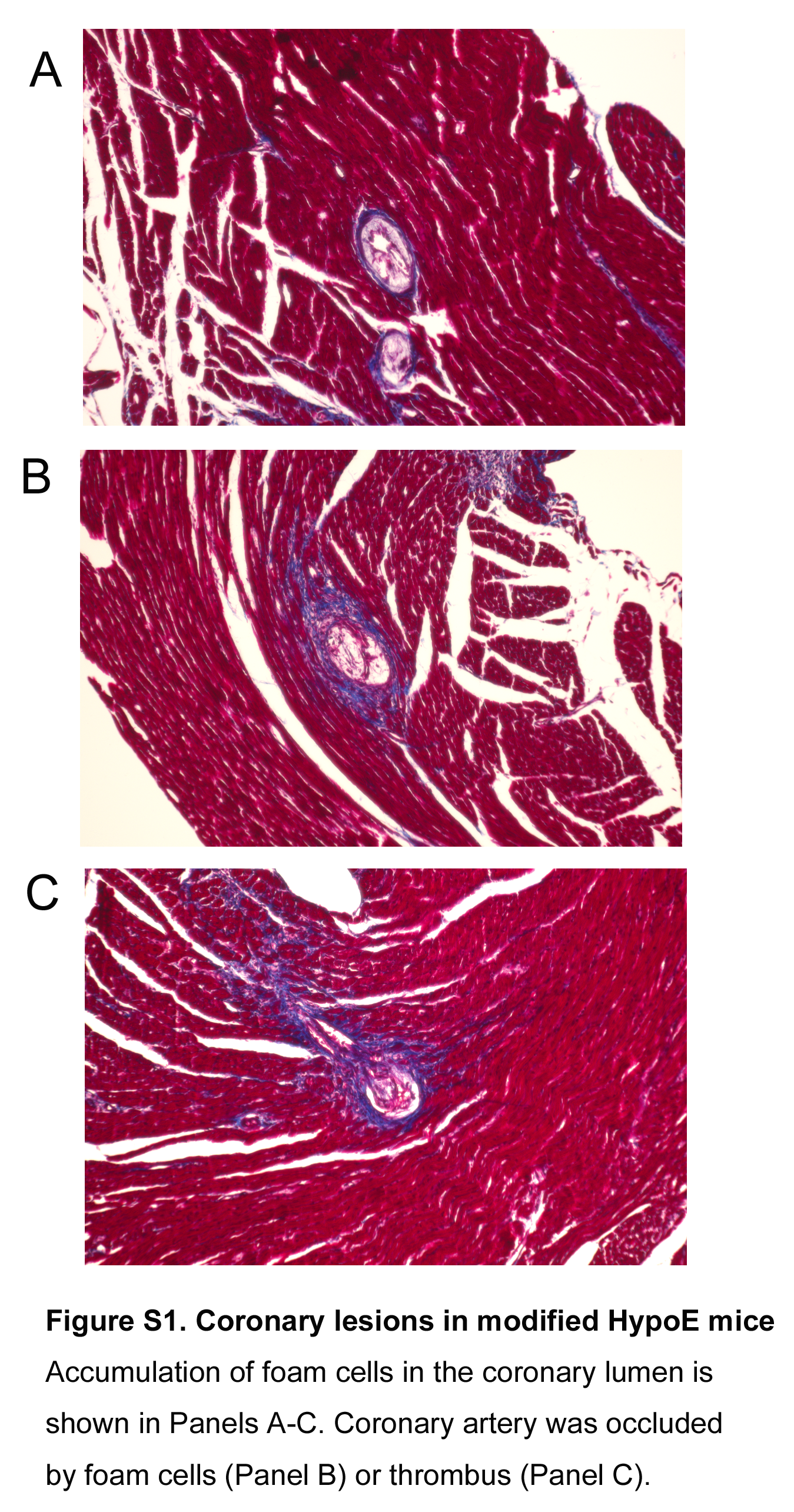

Supplement: Figure S1 — (TIF) [file pone.0070755.s001.tif]
